# Mapping Community-Engaged Implementation Strategies with Transgender Scientists, Stakeholders, and Trans-Led Community Organizations

**DOI:** 10.1007/s11904-023-00656-y

**Published:** 2023-04-04

**Authors:** Arjee Restar, Brian J. Minalga, Ma. Irene Quilantang, Tyler Adamson, Emerson Dusic, Leigh-Ann van der Merwe, Greg Millet, Danvic Rosadiño, Tanya Laguing, Elle Lett, Avery Everhart, Gregory Phillips, Rena Janamnuaysook, Pich Seekaew, Kellan Baker, Florence Ashley, Jeffrey Wickersham, Stephaun E. Wallace, Don Operario, Kristi E. Gamarel

**Affiliations:** 1 Department of Epidemiology, University of Washington School of Public Health, Seattle, WA USA; 2 Department of Social and Behavioral Sciences, Yale University School of Public Health, New Haven, CT USA; 3 Fred Hutchinson Cancer Center, Seattle, WA USA; 4 Department of Behavioral and Social Sciences, Brown University School of Public Health, Providence, RI USA; 5 Department of Health Policy and Management, Johns Hopkins University Bloomberg School of Public Health, Baltimore, MD USA; 6 Department of Biostatistics, University of Washington School of Public Health, Seattle, WA USA; 7 Social, Health and Empowerment Feminist Collective of Transgender Women of Africa, East London, South Africa; 8 The Foundation for AIDS Research, amfAR, amfAR, Washington, D.C USA; 9 LoveYourself Inc, Manila, Philippines; 10 DIOSSA Inc, Taguig, Philippines; 11 Computational Health Informatics Program, Boston Children’s Hospital, Boston, MA USA; 12 Department of Health Behavior and Health Education, School of Public Health, University of Michigan, Ann Arbor, MI USA; 13 Department of Medical Social Sciences, Northwestern University Feinberg School of Medicine, Chicago, IL USA; 14 Institute of HIV Research and Innovation, Bangkok, Thailand; 15 Tangerine Community Health Clinic, Bangkok, Thailand; 16 Department of Epidemiology, Mailman School of Public Health, Columbia University, New York, NY USA; 17 Whitman-Walker Institute, Washington, D.C USA; 18 Faculty of Law and Joint Centre for Bioethics, University of Toronto, Toronto, ON Canada; 19 Section of Infectious Disease, Department of Internal Medicine, Yale School of Medicine, New Haven, CT USA; 20 Global Health, University of Washington, Seattle, WA USA; 21 Department of Behavioral, Social, and Health Education Sciences, Emory Rollins School of Public Health, Atlanta, GA USA

**Keywords:** PrEP, Implementation, Transgender, Nonbinary, Gender-affirming care, HIV prevention

## Abstract

**Purpose of Review:**

Pre-exposure prophylaxis (PrEP) represents one of the most effective methods of prevention for HIV, but remains inequitable, leaving many transgender and nonbinary (trans) individuals unable to benefit from this resource. Deploying community-engaged PrEP implementation strategies for trans populations will be crucial for ending the HIV epidemic.

**Recent Findings:**

While most PrEP studies have progressed in addressing pertinent research questions about gender-affirming care and PrEP at the biomedical and clinical levels, research on how to best implement gender-affirming PrEP systems at the social, community, and structural levels remains outstanding.

**Summary:**

The science of community-engaged implementation to build gender-affirming PrEP systems must be more fully developed. Most published PrEP studies with trans people report on outcomes rather than processes, leaving out important lessons learned about how to design, integrate, and implement PrEP in tandem with gender-affirming care. The expertise of trans scientists, stakeholders, and trans-led community organizations is essential to building gender-affirming PrEP systems.

Designing gender-affirming PrEP systems goes beyond simply integrating gender-affirming care and PrEP services – it starts with partnerships with transgender scientists, stakeholders, trans-led community organizations, and community members at large.

## Introduction

A decade after Truvada as oral HIV pre-exposure prophylaxis (PrEP, tenofovir disoproxil/emtricitabine) was approved in 2012 by the U.S. Food and Drug Administration (FDA) for public use [1], multiple interventions and programs have demonstrated PrEP’s effectiveness to curb HIV incidence. Since then, three new major advancements were made in PrEP formulation and delivery. In particular, the Descovy formulation of PrEP (emtricitabine/tenofovir alafenamide) was evaluated for safety and efficacy in 2019. It received FDA approval and provided a second oral PrEP option for some adults and adolescents, but importantly, people assigned female at birth were excluded from the clinical research on Descovy, resulting in the FDA’s limited approval of Descovy for people assigned male at birth only [2]. More recently approved in 2021, Apretude (cabotegravir extended-release injectable suspension) became the first long-acting injectable (LAI) option that makes it possible to take PrEP every 2 months instead of in a daily pill form, but again, this option has transgender-specific limitations, as transmasculine people were not included in the clinical trials [3]. Lastly, as an alternative to daily oral PrEP, “on-demand” or 2–1-1 PrEP, which follows an arranged schedule of taking two Truvada pills 2 to 24 ho before sex, one pill 24 h after the first dose, and another pill 24 h after the second dose, is currently endorsed by the Centers for Disease Control and Prevention (CDC) to be taken only when “at-risk” for HIV [4], and with studies demonstrating its efficacy and effectiveness, yet largely conducted in cisgender populations [5–7]. These are monumental and promising advances in the HIV prevention landscape and the broader mission to improve the health of communities and end HIV/AIDS around the world. However, their reach is limited among communities of transgender and nonbinary (trans) people, due to the ongoing underrepresentation of these communities in PrEP research and implementation globally.

Trans populations are diverse and include communities of transfeminine, transmasculine, and nonbinary individuals [8, 9••].^1^ Trans communities face ubiquitous stigma, cissexism, and discrimination across all socio-ecological domains, including persistent structural efforts to delegitimize their gender identities and rights to access care, in addition to a dearth of comprehensive gender-affirming providers and services [10, 11]. These social and structural vulnerabilities place transgender and nonbinary communities at elevated risk for adverse behavioral and health outcomes, which further perpetuate to HIV inequities in this population [9••]. One recent study reported that transfeminine individuals, particularly those in the USA, have an estimated HIV prevalence of 14.1% [12••]. While existing data are scarce as a result of gender-blind surveillance systems [13], the limited data on transmasculine and nonbinary individuals who have sex with men show alarmingly high HIV incidence and prevalence as well [12••, 14]. Notably, the inequity in HIV prevalence is even more pronounced among minoritized ethnoracial groups [12••], within the trans community with estimates for HIV prevalence among Black, Indigenous, and Latine transgender women being substantially high [12••]. And community-led advocacy, research, and structural interventions that aim to promote HIV prevention interventions, including PrEP use, adherence, and persistence, must recognize the pressing need for equitable and transformative HIV prevention strategies that are rooted in and led by transgender and nonbinary communities [15–18].

## Inequities in Designing PrEP Studies with Trans Populations

Despite a wide diversity of gender identities and expression, PrEP research has largely focused on transfeminine adults. While this focus on transfeminine adults is likely due to the epidemiological evidence of high HIV burden among transfeminine individuals, it also reflects a paradigm in clinical research in which trans communities are perceived as ancillary to other key communities placed at risk [13, 19]. A 2021 scoping review focusing specifically on trans populations examined 667 HIV prevention articles that sampled at least one trans participant based on assigned sex at birth found that “38.5% subsumed transgender participants into cisgender populations (most frequently combining trans women with cisgender men who have sex with men), 20.4% compared transgender and cisgender participants, and 41.1% focused exclusively on transgender women” [20••]. While this review is aimed at comprehensively providing an overview for all trans groups, data extracted were only able to delineate the inclusion of transfeminine adults in HIV studies and were unable to distinguish transmasculine and nonbinary people. To date, few reviews have been conducted to delineate the participation of either transmasculine or nonbinary individuals in HIV or PrEP studies [21], reflective of the erasure and lack of gender-inclusive approaches in PrEP studies and in the broader HIV landscape.

While there have been improvements in including transfeminine adults in PrEP and HIV research and programming, glaring intersecting gender and racial inequities remain. The inclusion of transgender and nonbinary individuals in implementation research, clinical trials, and community engagement has remained at best, scant, despite the growing number of transgender and nonbinary communities in the USA [12••]. Globally, particularly in the global south where community engagement and leadership with trans advocates have gained more prominence, PrEP programming and HIV surveillance systems are only now beginning to implement changes and correct systematic practices that subsumed trans populations into other key populations [22, 23]. And while PrEP studies and HIV surveillances have relied on sex assigned at birth as part of eligibility criteria as well as to identify trans individuals in primary data collection and in existing databases, such an approach not only disregards the diversity in trans identities but also complicates and furthers data misrepresentation rendering some to the point of invisibility, where the true burden of need remains unknown. This further limits any understanding of ethno-racial differences within subgroups of trans communities, where health inequities [24••], including HIV has been documented [25]. Trans and HIV scholars have recommended designing studies with self-reported demographic data on gender identity or trans status to be the preferred method for measuring and identifying trans populations in studies and surveillance systems, and for conducting research with trans communities [9••, 26••].

## Persistence for Gender-Affirmative PrEP Systems and Trans Engagement

Advancements in PrEP have scarcely included, prioritized, or positioned trans people as stakeholders, as scientists, as partners with trans-led community-based organizations, and as we noted above, not even as research participants. This exclusion is due to the pervasiveness of cissexism and cisnormativity that leads to erasure in research and public health programming at-large that researchers—both cis and trans—reinforce and uphold [18, 27••]. Many have begun working to heal relationships between trans communities, HIV research, and scientific communities through community-engaged research and programmatic interventions [28–34], but much more work is needed [35]. Several trans and nonbinary scientists and community scholars working within spaces of HIV prevention have recurrently advocated for HIV prevention research and programming to be conducted *with* them across all stages of research development, implementation, and dissemination [17, 32].

Specifically, there has been a persistent demand by trans people for PrEP programmers to design and implement gender-affirmative PrEP systems [16]. Gender-affirmative PrEP systems necessitate for trans people to receive the necessary PrEP care continuum services [36] while also highly valuing and ensuring that trans people’s gender identity and treatment goals are medically, socially, and structurally recognized and supported—to maximize the benefits of PrEP within trans communities directly [15, 37]. In the context of HIV care, this includes the critical and comprehensive integration of HIV care with gender-affirming health services, which has been shown to improve engagement and retention for achieving viral suppression [38–40]. There have been some efforts to implement and evaluate gender-affirmative PrEP programs in existing healthcare settings [29, 41]; however, additional efforts are warranted to address PrEP barriers across the personal/biological, social, structural socio-ecological levels. Specifically, the transformation of health care settings towards gender-affirming warrants the implementation of policies in different geographic locales that eliminate HIV criminalization as well as bans or restrictions to legal affirmation, such as name and gender marker changes both in government-issued identification documents and electronic medical records [42]. For example, the Trans Renaming Project in Detroit, Michigan, a community-led initiative, is aimed at improving access to legal affirmation and address social and legal barriers that contribute to HIV inequities and inaccessibility to PrEP and HIV prevention services for trans communities locally [34, 43].

At the biomedical level, formative research has been conducted with transfeminine adults to examine PrEP efficacy along with gender-affirming hormones. Specifically, concerns for lowered efficacy and drug-drug interactions between PrEP and gender-affirming hormones have been documented, both from the perspectives of providers and trans community members [44, 45]. To address this concern, several pharmacological studies have examined the impact of PrEP on hormones and vice versa [45–47]. One pharmacological clinical trial with Thai transfeminine adults offers clarity showing that estrogen hormones do not lower PrEP efficacy in a clinically significant way and that estrogen levels were not affected by PrEP [48]. In a recent double-blind noninferiority trial with transfeminine adults, findings demonstrated that blood-level concentration of two types of PrEP formulation (emtricitabine and tenofovir alafenamide (F/TAF) versus emtricitabine and tenofovir disoproxil fumarate (F/TDF)) remained efficacious and were not negatively impacted by gender-affirming hormones [49]. However, studies on the efficacies and effectiveness of taking both gender-affirming hormones with other important PrEP modalities such as long-acting injectable PrEP and 2–1-1 are underexamined. Despite this limitation, some expert opinions convey that there are no expected drug-to-drug interactions between gender-affirming hormones and PrEP, and though more studies are needed, authors recommended to continue offering PrEP for trans communities given studies showing the reach of protective concentrations [45]. Scholars have also noted the need to train PrEP providers to utilize gender-affirming rhetoric and adopt a personalized medicine approach when prescribing gender-affirming care with PrEP [50]. This approach not only allows for providers to monitor and ensure that PrEP and hormone blood levels remain safe but also to align and meet patients’ PrEP and other HIV services needs with their gender affirmation goals [50].

At the clinic-level, a select few US- and international-based demonstration projects have shown some promising successes in implementing the integration of gender-affirming care with PrEP services, with the majority primarily designed to increase PrEP uptake among transfeminine adults in community-based health clinic settings. For example, researchers at Callen Lorde Community Health Center in New York City, an LGBTQ-focused health center that specializes in the integration of PrEP, gender-affirming care, as well as insurance and payment services, recently published a longitudinal study that showed PrEP adherence was high (greater than 90%) at 3 and 6 months after initiation both in self-report and urine assay data collection among 80% of the 100 enrolled transfeminine patients [51]. In Atlanta, a patient-centered PrEP program designed with a co-located gender clinic offering affordable comprehensive primary care, gender-affirming hormone, and mental health services showed high rates of linkage to PrEP care, prescription, and initiation among transfeminine participants. In Thailand, key population-led health service programming that offers same-day PrEP and is delivered by trained key population community health workers, including transgender lay providers, contributed to 82% current Thai PrEP users and highlighted adherence and retention as high-priority research areas for scale-up of the program [29, 52]. In addition, the Tangerine Clinic in Thailand introduced the “Integrated Trans Model,” which was disseminated to three other Asian countries using implementation strategies informed by community leaders and resulted in PrEP linkages ranged from 20 to 27% [53]. In the Philippines, community-based organizations and trans community leaders are building out grassroots community outreach, infrastructure, and organizational capacity for ongoing gender affirming care, PrEP and HIV research clinics, including offering in-person and telehealth services, training health care workforce, organizing community events, and building formal coalitions such as the Philippine Professional Association for Transgender Health to improve gender-affirming care as well as combat recent surges in HIV incidence [54]. Similarly in South Africa, scaling up of PrEP clinics are underway with aims to provide comprehensive care in addition to PrEP for transgender women, with results under review at the time of this review [55]. To our team’s knowledge, only one study in California has been inclusive of transmasculine and nonbinary communities and demonstrated high levels of PrEP initiation across gender identities; however, gender differences were found in adherence to daily oral PrEP such that transfeminine adults were more likely to have protective drug levels compared to transmasculine and nonbinary adults [41]. As such, future research is warranted to develop and implement gender-affirming PrEP programs that addresses barriers across the PrEP cascade.

## Recommendations for Community-Engaged Gender-Affirming PrEP Systems

**Fig. 1:**
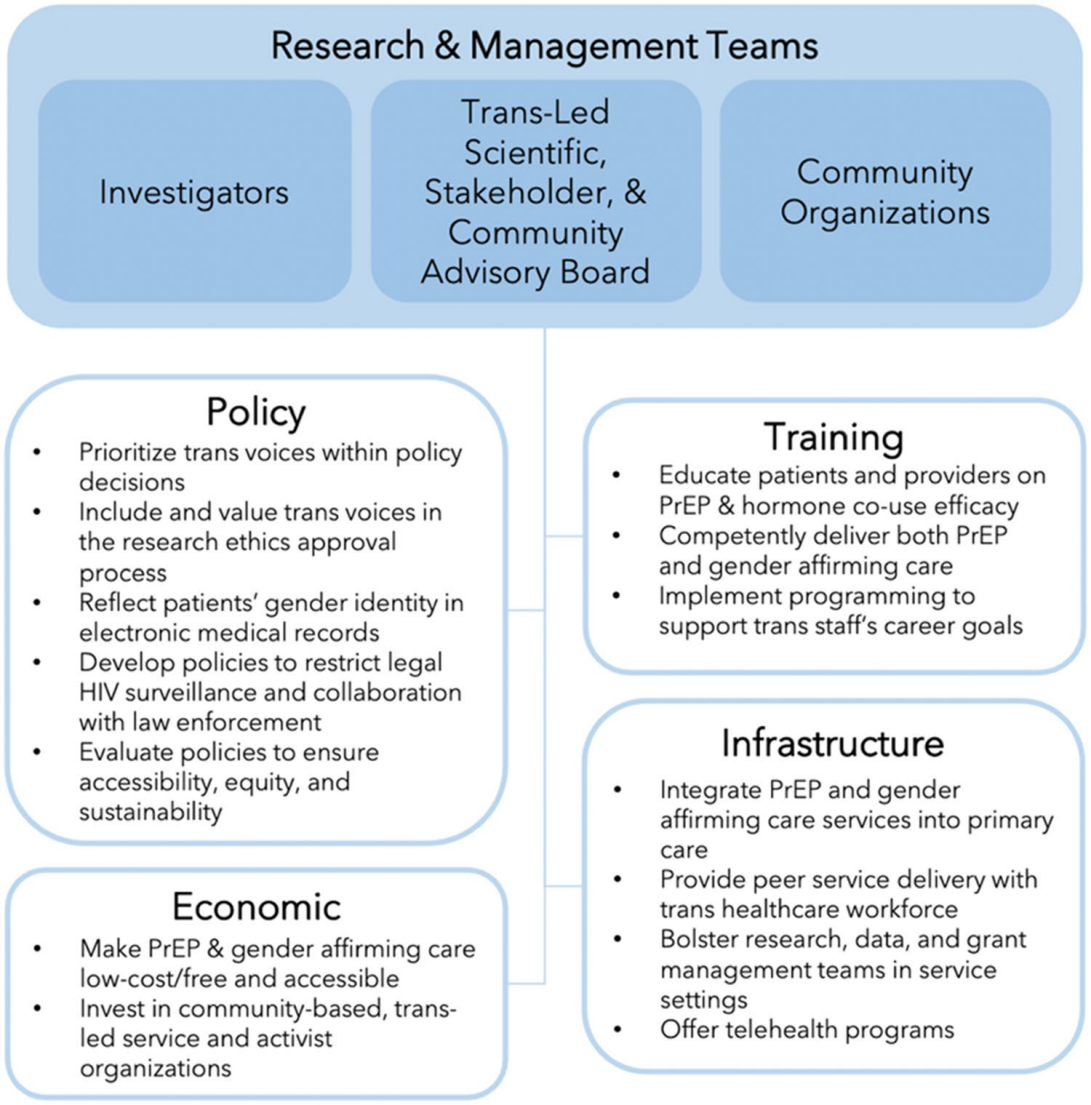
Community-engaged implementation strategies as applied to gender-affirmative PrEP systems

Envisioning what gender-affirming PrEP systems look like across other socio-ecological levels remains underdeveloped. Published PrEP studies typically report on outcomes rather than processes. As a step to fill this gap, we turn to documented strategies drawn from the literature, and when possible, leaned on our expertise as trans stakeholders, leaders of trans-led community organizations, and as communities of trans and cis scholars in the fields of trans health and HIV prevention to map (in Fig. 1) and synthesize the following recommendations (in Table 1).

**Table 1:** Recommendations for community-engaged gender-affirming PrEP systems

**Infrastructure**	
• Create leadership roles and trans-led scientific, stakeholder and community advisory board (TSSAB) governing bodies	Position trans scientists, stakeholders, and community members in leadership roles that provide equal partnerships in programmatic decision processes. Additionally, create advisory board memberships with trans-community members in which members will have the influence to co-design collaboratively to refine programmatic procedures, protocols, and communication strategies, infusing the program with community knowledge and expertise throughout the design, execution, and scale-up phases [56]
• Design PrEP services holistically with high-quality gender-affirming care	Given that transgender and nonbinary people have other competing health needs other than HIV prevention, integrate high-quality gender-affirming services with PrEP/HIV prevention services to remove structural barriers that keep these care services fragmented. Integrate and develop gender-affirming care and HIV prevention as part of primary care services to optimize meeting unmet health needs
• Integrate PrEP programs with existing state and federally funded Social Determinants of Health (SDOH)-related services and support	Social determinants and factors such as housing, employment, income, transportation, education, and health literacy, health-harming legal needs, and insurance play an important role in uptake and maintenance of both gender-affirming care and PrEP [57–61]. Situate gender-affirming and HIV prevention and needs in the context of programs designed to address social determinants of HIV
• Employ peer-delivery program structure with trans staff and health care workforce	Peer-based health programs have been shown to improve the quality of health service delivery among patients [31, 38, 61, 62]. Employing trans individuals as part of key workforce personnel (e.g., peer health navigators, providers) has been shown to positively impact quality and retention in health services [23, 29, 34, 63–65]
• Bolster organizational and facility capacity to carry out research and data analyses	Programs perform best when informed by real-time data. However, most community-based organizations and facilities do not have the resources for developing and sustaining a research and data arm, let alone a dedicated data team. As such, it is vital to develop capacity and the allocation of resources in these areas that allow for in-house data analyses and continued, real-time monitoring of PrEP and gender-affirming services. Additional support for liaising with higher level data collection agencies is also necessary to facilitate data harmonization and aggregation for high-quality trans population health surveillance. There is an ethical responsibility for academics working in this area dedicate their time to ensure continuity of research and data analyses. This will also allow for better data on gender identities in health care surveillance tools, making true understanding of the population size and health impacts of PrEP and gender affirming care possible
• Develop research and management teams of trans-led community-based organization and facilities	Federal- and state-sponsored funders have called for the inclusion of community partners in HIV prevention research endeavors; however, there are a multitude of systemic barriers to equitable community-academic partnerships, such as setting up eRA Commons accounts with partners who do not have the research infrastructure or resources. More importantly, there is a need to simultaneously support trans-led community-based organizations and partner facilities to develop their own research and grant management, so that they too can be competitive to apply for federal and state-sponsored grants and programs as lead principal investigators and receive investments directly for maximum utility of funds. University and other institution-led research often include grant-related financial bureaucracies that cut allocation of resources short from community partners. The allocation of support and resources will allow community stakeholders and trans-led organization to set research questions and agenda about gender-affirming PrEP systems and lead impactful research projects
• Infuse lessons learned from COVID-19 telehealth programming for both hormones and PrEP services	Drawing lessons learned from the COVID-19 response, telehealth has emerged as a critical programmatic tool to deliver and facilitate health services, especially among trans populations residing in areas where health care continues to be fragmented by geography (e.g., PrEP/gender affirming care deserts) and/or anti-trans health care policies [66–68]. Leveraging telehealth, including remote clinical visits, at-home lab collection services, and local or delivered pharmacy services, can increase access to gender-affirming PrEP programs for trans populations [54, 69]
**Training and education**	
• Develop health literacy and communication programs highlighting the efficacy of PrEP and hormones	Given recent evidence showing continued efficacies of both PrEP and hormones when taken con-currently, we recommend developing and creating theory- and evidence-based education interventions and/or health literacy programs with curriculum materials and health communication strategies focused on concerns and misinformation about PrEP and hormones among providers and transgender and nonbinary patients to help manage decisions about care. Rather than stigmatizing risk behaviors of transgender people, utilize gain-framed counseling approach to encourage clients to focus on protection and healthy behaviors to increase PrEP and gender affirming care uptake [70]
• Train providers on PrEP and high-quality gender-affirming care	Providers working within a gender-affirming PrEP system must be competent in both PrEP and high-quality gender affirming care. PrEP providers can be trained on gender-affirming care, and vice versa [41, 71]. This includes ensuring providers are well-versed in adopting a personalized medicine approach and gender affirmative rhetoric to meet patients where they are and where they want to be based on their hormone goals as well as HIV prevention goals [26••, 50, 72]. It also includes educating providers to understand some of the salient reasons and motivations for both gender affirming and HIV prevention goals and how they may change over time, while valuing continued routine check-ups/monitoring to ensure pharmacological safety levels of both medicines
• Develop training programs to support staff professional goals	As trans individuals become key personnel in gender-affirmative PrEP systems, provide training pipelines and opportunities over time for professional growth in areas of health care management, communication, leadership, research, policy, and education
**Economic**	
• Offer PrEP and gender affirming care with affordable to free payment systems	It is well documented that cost is a barrier to both PrEP and gender affirming care uptake. This includes cost of medication, transportation to/from clinics, in visit fees, and lab fees. Offering PrEP and gender affirming hormones with low-cost to free payment system addresses these barriers by leveraging federally and state-funded financial PrEP programs, as well as by expanding insurance coverage for both medications and fees associated. Additionally, consider incentivization strategies to promote uptake and adherence and offset economic barriers
• Continued investments in community-based gender-transformative efforts, particularly for trans-led organizations	Root causes of gender inequities are tied to HIV inequities experienced by many trans communities and are intrinsically linked to systemic ethno-racism. Providing committed investments to support transgender-focused and community-based organizing efforts curated by partnered trans-led organizations that aim to dismantle system of intersectional oppressions are integral to prioritizing and safeguarding the health and lives of trans communities
**Policy**	
• Center and value trans voices in policy decisions	Default to having trans people make evidence-based policy decisions about trans health and HIV-related policies. Include all transgender and nonbinary scientists, stakeholders, and community members across all levels of policy-developments and decisions
• Reevaluate PrEP and gender-affirming care policies to ensure optimal accessibility	Reassess and/or propose improvements to current policies on PrEP and gender affirming care to be guided by principles of accessibility, equity, affordability, sustainability, and antidiscrimination. Remove policies and referral assessments reinforces gatekeeping of gender affirming care [73]
• Include and value trans voices in the research ethics approval process	Ensure the involvement of transgender and nonbinary people in ethics approval for research on PrEP and HIV prevention
• Align policies with legal affirmation	Recognizing the health-harming legal needs experienced by trans people that contributes to HIV inequities, implement policies that eliminate HIV criminalization and systems which disproportionately surveil/police people of color create restrictions to legal affirmation (e.g., legal gender marker and name changes) on records
• Develop policies to restrict HIV surveillance and collaboration with law enforcement	Fear that data collected by researchers and organizations could be used to criminalize service-users for HIV non-disclosure creates a barrier to access [42]. Researchers and service providers should develop policies that reduce the possibility of surveillance and minimize collaboration with law enforcement

## Conclusion

Despite the development of multiple PrEP formulations now available for delivery, trans people experience suboptimal benefits due to low community engagement resulting in inequitable access and uptake of this medication. The expertise of trans people has not been adequately incorporated into the design of PrEP education and promotion campaigns. The complex health needs of trans communities have not been sufficiently consulted in the implementation of PrEP delivery systems. Consequently, trans communities continue to suffer disproportionately from the unnecessary and preventable burden of new HIV infections. This inequity must be remedied.

Meaningful and sustained investments in partnerships with trans community members are essential to mitigating widening inequities of HIV burden that disproportionately affect trans people worldwide. Specific actions are as follows: (i) recognize transmasculine and nonbinary individuals (i.e., not just transfeminine individuals) as priorities for PrEP programming; (ii) bring visibility to similarities and differences in gender-affirmation needs across the spectrum of gender minority groups by distinguishing gender-inclusive and gender-specific approaches in designing PrEP delivery systems (19); (iii) address social determinants of HIV within trans populations including—but not limited to—the role of employment, insurance, housing, legal factors that shape the ability for trans people to achieve optimal health; (iv) include trans scholars and scientists in positions of leadership in future PrEP research and programming efforts.

## Footnotes

1 Transfeminine is a gender identity that describes people who were assigned male sex at birth and now with a gender identity within the transfeminine spectrum (e.g., trans women, women), and transmasculine is a gender identity that describes people who were assigned female sex at birth and now with gender identity within the transmasculine spectrum (e.g., trans men, men). And nonbinary describes people whose gender identities do not conform with the gender binary.
